# Quantification of Polystyrene Uptake by Different Cell Lines Using Fluorescence Microscopy and Label-Free Visualization of Intracellular Polystyrene Particles by Raman Microspectroscopic Imaging

**DOI:** 10.3390/cells13050454

**Published:** 2024-03-05

**Authors:** Amelie Roth, Astrid Tannert, Nadja Ziller, Simone Eiserloh, Bianca Göhrig, Rustam R. Guliev, María José Gonzalez Vazquez, Max Naumann, Alexander S. Mosig, Sven Stengel, Astrid R. R. Heutelbeck, Ute Neugebauer

**Affiliations:** 1 Leibniz Institute of Photonic Technology, 07745 Jena, Germany; 2 Occupational, Social and Environmental Medicine, Jena University Hospital, 07747 Jena, Germany; 3 Center for Sepsis Control and Care and Department of Anaesthesiology and Intensive Care Medicine, Jena University Hospital, 07747 Jena, Germany; 4 Institute of Biochemistry II, Jena University Hospital, 07747 Jena, Germany; 5 Department of Neuropediatrics, Jena University Hospital, 07747 Jena, Germany; 6 Institute of Physical Chemistry and Abbe Center of Photonics, Friedrich Schiller University, 07743 Jena, Germany

**Keywords:** microplastics, cellular update, THP-1 macrophages, CaCo-2 cells, A549 cells, 3D Raman imaging, airborne microplastics, environmental sample

## Abstract

Environmental pollution caused by plastic is a present problem. Polystyrene is a widely used packaging material (e.g., Styrofoam) that can be broken down into microplastics through abrasion. Once the plastic is released into the environment, it is dispersed by wind and atmospheric dust. In this study, we investigated the uptake of polystyrene particles into human cells using A549 cells as a model of the alveolar epithelial barrier, CaCo-2 cells as a model of the intestinal epithelial barrier, and THP-1 cells as a model of immune cells to simulate a possible uptake of microplastics by inhalation, oral uptake, and interaction with the cellular immune system, respectively. The uptake of fluorescence-labeled beads by the different cell types was investigated by confocal laser scanning microscopy in a semi-quantitative, concentration-dependent manner. Additionally, we used Raman spectroscopy as a complementary method for label-free qualitative detection and the visualization of polystyrene within cells. The uptake of polystyrene beads by all investigated cell types was detected, while the uptake behavior of professional phagocytes (THP-1) differed from that of adherent epithelial cells.

## 1. Introduction

Polystyrene (PS) is a synthetic polymer made from monomers of the aromatic hydrocarbon styrene. General-purpose polystyrene is clear, hard, and brittle. Polystyrene is one of the most widely used plastics, with the scale of its production being several million tons per year [1]. It is ubiquitous in our daily lives, often found in the form of disposable items like foam cups, food containers, packaging materials, and single-use cutlery. Polystyrene from discarded products and materials can break down into small particles over time due to weathering and mechanical abrasion. Exposure to UV radiation causes polystyrene to rot and become brittle so that it then tends to crack. The smaller particles are called microplastics when their size varies between 0.0001 millimetres (mm) and smaller than 5 mm (recommendation on the definition of a nanomaterial, 2011/696/EU). Since plastic is hardly completely degradable, it remains in the environment for an indefinite period of time. Tiny airborne particles can be carried by wind and become part of the atmospheric dust. They can also accumulate in all biotopes, including the oceans, threatening wildlife and habitats [2,3]. There is growing awareness that the presence of microplastics in the environment can lead to the ingestion of microplastics through the digestive and respiratory systems [4]. Associated potential health risks are still an area of ongoing research [5,6,7]. Therefore, elucidating the interaction between nanoparticles and living cells has become a central focus of research. Cellular uptake is a pivotal process that underpins the efficacy and safety of nanoparticle-based applications.

In our study, we have chosen three different cell lines, which can be considered as model systems for potential barriers that nanoparticles encounter upon contact with the human body. The A549 cell line is a model for the lung epithelium being a barrier during inhalation, the CaCo-2 cell line mimics gut epithelial cells, which poses the barrier after oral uptake, and the THP-1 cell line represents monocytes/macrophages as part of the immune system. The uptake of nanoparticles is expected to take place by energy-dependent endocytosis involving the actin polymerization. While all cell types are able to use pinocytosis as an uptake process, phagocytosis seems to be restricted to specialized professional phagocytes including macrophages, neutrophils, and monocytes [8].

The diversity of plastics complicates the qualitative (i.e., identification of the plastic material) and, especially, the quantitative (i.e., how many microparticles are measured) analysis of microplastics. At present, there is no validated, i.e., generally accepted and tested, method for the identification and quantitative analysis of microplastics. Different analytical approaches for the determination and quantification of microplastics are currently being discussed [9,10,11,12,13]. In general, microplastics can be identified by physical characterization (microscopy) followed by chemical characterization (spectroscopy) for plastic confirmation [1,14]. Among the spectroscopic methods, Raman spectroscopy and Fourier transform infrared spectroscopy (FTIR) proved to have high potential as they provide characteristic polymer spectra with which the plastic particles can be identified using a reference library [15,16,17].

Our investigation seeks to provide quantitative insights into cellular uptake by applying powerful imaging techniques. Confocal fluorescence microscopy, a well-established method with high sensitivity and diffraction-limited spatial resolution, allows for quantification and visualization of internalized nanoparticles in 3D, providing valuable insights into the ease of uptake and particle distribution within the different cell lines [18]. In addition, we present Raman spectroscopic imaging as a powerful, label-free, and non-invasive qualitative visualization method of intracellular polystyrene particles. This approach does not require any fluorescence tags and allows for an identification of the chemical identity of the nanoparticles directly within intact cells. Similarly, the uptake of fluorescently labelled microplastics has been studied previously in whole jelly fish using both confocal microscopy, TEM, and Raman microscopic imaging [19].

## 2. Materials and Methods

### 2.1. Nanoparticles, Cell Culture, and Incubation

For fluorescence imaging, Dragon Green-labelled fluorescent polystyrene beads (diameter: 200 nm, 1.02% *w*/*v*, Bangs Labratories Inc., Fishers, IN, USA) were used. For the Raman study, plain polystyrene beads without fluorophore (diameter: 220 nm, 5% *w*/*v*, Spherotec, Lake Forest, IL, USA) were used.

Adherent A549 cells and THP-1 suspension cells were cultivated at 37 °C with 5% CO_2_ in RPMI Medium 1640 (1×) with GlutaMAX^TM^ (Gibco, Thermo Fisher, Darmstadt, Germany) supplemented with 10% FCS (Gibco, Thermo Fisher, Darmstadt, Germany) and 1% Penicillin-Streptomycin (Pen Strep 10,000 U/mL, Gibco, Thermo Fisher, Darmstadt, Germany). In addition to culture media, THP-1 cells were incubated for 24 h with 100 ng/mL of Phorbol myristate acetate (PMA, InvivoGen, Toulouse, France) for differentiation into macrophages and adherence onto CaF_2_ slides. Adherent CaCo-2 cells were cultivated at 37 °C with 5% CO_2_ in DMEM 4.5 g/L (1×) with GlutaMAX^TM^ (Gibco, Thermo Fisher, Darmstadt, Germany) supplemented with 10% FCS, 1% NEA (100×) (Gibco, Thermo Fisher, Darmstadt, Germany), 1% pyruvate (1×, 100 mM) (Gibco, Thermo Fisher, Darmstadt, Germany company), and 1% Penicillin–Streptomycin. Adherent and suspended cells were treated with 0.75% dimethyl sulfoxide (DMSO, Carl Roth, Karlsruhe, Germany) before bead stimulation following the recommendations of the manufacturer of the polystyrene beads to keep beads stable in suspension. The untreated control cells were handled similarly.

For fluorescence measurements, various numbers of cells (A549: 1500–12,000 cells/well, THP-1: 25,000 cells/well, CaCo-2: 2000 cells/well) were placed in an eight-well coverslip chamber slide with a growth area of 0.8 cm^2^/well (Sarstedt, Nümbrecht, Germany). After 24 h, nanoparticles were added in the respective concentrations, i.e., 25 µg nanoparticles/well (31.25 µg/cm^2^), 12.5 µg nanoparticles/well 15.625 µg/cm^2^), 6.25 µg nanoparticles/well (7.81 µg/cm^2^), or no nanoparticles as controls and further incubated for 24 h or 72 h.

For Raman measurements, 40,000 THP-1 cells were placed in six-well plates containing an 8 mm × 12 mm CaF_2_ slide (Crystal GmbH, Berlin, Germany) as a substrate. Similar to sample preparation for the fluorescence measurements, nanoparticles were added after 24 h to the well in the respective concentrations, i.e., 50 µg of nanoparticles/well (appr. 5.6 µg/cm^2^). 

All experiments were performed in triplicates. 

After incubation with nanoparticles for 24 h or 72 h, cells were washed three times with DPBS (1×) (Gibco, Thermo Fisher, Darmstadt, Germany) followed by fixation with 4% paraformaldehyde (PFA, Merck KGaA, Darmstadt, Germany) for 15–30 min and again washed three times and stored until further use.

### 2.2. Fluorescence Labeling

After fixation at the end of the uptake experiment, cells were additionally labeled to reveal cell morphology. Therefore, the cells were permeabilized using 0.1% Triton^®^ X-100 (Carl Roth, Karlsruhe, Germany), followed by blocking with Immunoblock (Carl Roth, Karlsruhe, Germany) in a 1:10 dilution for 15–30 min at room temperature. Subsequently, the actin cytoskeleton was stained using 2 rxn/mL Phalloidin 555-I (Abnova Corporation, Taipei City, Taiwan) for one hour at room temperature and washed, while nuclei were counterstained using DAPI (AppliChem, Darmstadt, Germany) at a concentration of 0.2 µg/mL at room temperature. Some control chambers remained unstained to reveal the amount of cellular autofluorescence in the different channels.

### 2.3. Fluorescence Measurements

Fluorescence measurements were conducted using a confocal laser scanning microscope CLSM 780 meta (Carl Zeiss, Jena, Germany) for measurements of the THP-1 cells and a CLSM 980 (Carl Zeiss, Jena, Germany) for characterizing the A549 and CaCo-2 cells. Images were acquired using a Plan Apochromat 20×/0.8 NA objective (Carl Zeiss, Jena, Germany) for overview images of four-to-six fields of view (FOV), each about 350 µm × 350 µm in size, whereas a Plan Apochromat 40×/0.95 NA objective (Carl Zeiss, Jena, Germany) was used for additional detailed images. Dragon Green polystyrene beads were excited with 488 nm and detected in the range of 490–571 nm and I555 phalloidin was excited using a 561 nm laser and detected in the range of 562–615 nm, while DAPI was excited with 405 nm and detected in a range of 409–493 nm, additionally detecting bright field to reveal cellular localization. To avoid channel crosstalk, all channels were recorded sequentially. Images were acquired in different focal planes (z-stack) at a distance of 1 µm and covering the entire height of the cell layer.

### 2.4. Analysis of Fluorescence Data

Data analysis was conducted using Fiji ImageJ, 1.54f, National Institute of Health, Bethesda, MD, USA [20], where a z-projection was performed first, followed by channel splitting. Only the two channels of the Dragon Green beads and the DAPI-stained nuclei were of interest for determining the number of bead pixels per cell. Staining with phalloidin was used to distinguish individual cells from each other. To exclude any fluorescence from remaining extracellular beads, which were occasionally observed after washing, the cell area was determined using either the fluorescence recorded in the IF555 channel (fluorescent actin cytoskeleton and cellular autofluorescence) or, if not sufficient, the additionally recorded bright field images. In the latter case, the images were first subjected to a contrast enhancement, followed by a canny edge detection, maximum filter, and binary morphology operations (closing and opening) [21]. Images were segmented accordingly, and any fluorescence originating from non-cellular areas was omitted from subsequent analysis.

In the DAPI image, a Gaussian blur filter with σ = 3 is first applied to the image to achieve an accurate delineation from the background by smoothing the edges of the nuclei. In the following step, a threshold was manually set where the mask corresponds to the original image capture. This is shown more clearly in Supplementary Figure S1. In addition, some cell nuclei can be very close to each other, which made it difficult to delineate them accurately. To counteract this problem, watershed segmentation was also performed afterwards. Now, the cell nuclei were counted by using the “Analyze Particles” function. A minimum particle size of 20 µm^2^ was entered for THP-1 cells and a minimum size of 10 µm^2^ for A549 and CaCo-2 cells. The circularity was set to 0.0–1.0 for all three cell types. The average counted cell number per one mm^2^ for the different cell lines and time points are provided for the three replicates in Supplementary Table S1.

Due to the small size and the fact that beads could no longer be distinguished from each other as soon as they are close to one another, the pixels, in which a nanoparticle fluorescence signal is recognizable, were counted. Therefore, we calculated the area covered by pixels with green fluorescence above a certain threshold (pixels containing nanoparticle fluorescence) and related this area to the number of cell nuclei in the image. We assumed that a higher area covered by nanoparticles correlates with a higher total particle number. As a first step, a threshold was set on the image of the green fluorescent beads (Supplementary Figure S1). The threshold value was determined using the images of cells not incubated with nanoparticles (in order to take possible autofluorescence into account) as reference. The exact high of the threshold was manually adjusted to ensure inclusion of complete bead fluorescence while excluding unspecific autofluorescence by comparing the mask with the recorded image. The image is then set to binary and the number of pixels above the threshold is determined by calculating the mean grey value, normalizing this to one (division by 255 for an 8-bit image) and multiplying it by the number of pixels in the image (for visualization, see also Supplementary Figure S1):

(1) $$\frac{size\;image\;scan\;in\;pixel\;}{255}\times mean\;gray\;value\;bead\;pixel=amount\;of\;pixel\;that\;shows\;PS\;bead\;signal$$

The area covered with bead fluorescence can then be related to the number of cells as calculated from the number of nuclei in the field of view:

(2) $$\frac{amount\;of\;pixel\;that\;shows\;PS\;bead\;signal}{amount\;of\;cell\;nuclei\;in\;image\;scan}=number\;of\;beadpixel\;per\;one\;cell$$

Statistical significance between detected bead pixels per cell at different concentrations of nanaoparticles was tested using two-sided unpaired *t*-test. The concentration-depended increase of bead pixels per cell was fitted using linear regression and confidence intervals were calculated using “lm” function from standard R package “stats”.

### 2.5. Raman Measurements

Raman measurements were performed using an upright Raman microscope (CRM alpha 300, WiTec GmbH, Ulm, Germany). Raman spectra were excited using a laser wavelength of 532 nm, which is focused onto the sample using a 60× water-immersion objective (NA 1.0, Nikon, Melville, NY, USA), providing 15 mW in the sample plane. Inelastically back-scattered light was collected and guided with a 25 µm-diameter fiber onto a grating with 600 grooves/mm and recorded using a charge coupled device; DU401A BV-532 (ANDOR, 1024 × 127 pixels, cooled to −60 °C). Cells stimulated with polystyrene beads (ø 220 nm) for 24 h were characterized by Raman imaging in different z planes. Image size was chosen to fit the whole cell or several adjacent cells (area of 25 µm × 25 µm or 60 µm × 60 µm). Step size was 0.5 µm for the smaller image scan and 1 µm for the larger scans. Integration time per pixel was 1 s. Daily performance check of the device was performed using a standardized silicon sample and 4-acetamidophenol.

### 2.6. Analysis of Raman Data

Evaluations of Raman spectra and the respective images were performed using R (version 4.2.2), R foundation, Vienna, Austria, R Studio (version 1.4) IDE, Posit, Boston, MA, USA, in-house written R scripts, and the following packages: dplyr [22], hyperSpec [23], ggplot2 [24], and unmixR [25].

First, Raman spectral data were pre-processed by removing spikes using algorithm described in [26], correcting baseline using SNIP algorithm, normalizing intensities by mean, and cutting out silent spectral region 1800 cm^−1^ till 2700 cm^−1^. After the pre-processing, Raman hyperspectral images were unmixed, i.e., each pixel was presented as a linear mixture of pure components (endmembers) unknown beforehand. N-FINDR algorithm [27] was used for endmember extraction and non-negative least squares (NNLS) for calculating corresponding abundances. Number of endmembers was chosen manually by interactively increasing the number and examination of corresponding endmembers. 

## 3. Results

The successful cellular uptake of polystyrene nanoparticles was confirmed using two complementary biophotonic methods, namely confocal fluorescence microscopy and Raman spectroscopic imaging. Fluorescence microscopy enables the analysis of several cells in a short time but requires fluorescently labeled nanoparticles. Raman spectroscopy, on the other hand, can visualize polystyrene nanoparticles within intact eukaryotic cells without the need for any label in three dimensions. In the following, results from both imaging approaches are presented.

### 3.1. Quantification of Polystyrene Nanoparticle Uptake by Different Cell Lines

Exemplarily for the different entry pathways of nanoparticles from the environment into the human body, A459 cells were chosen as a model of lung epithelium cells mimicking the barrier during inhalation, and CaCo-2 cells were chosen as a model for gut epithelial cells mimicking the barrier during oral uptake. In addition, THP-1 cells were included in the study as representatives of immune cells, particularly monocytes/macrophages, which act as professional phagocytosing cells. Their task in the body is to engulf and digest foreign particles, such as pathogens like bacteria or cells infected with viruses. Thus, it is expected that they are also actively taking up polymeric nanoparticles.

**Figure 1:**
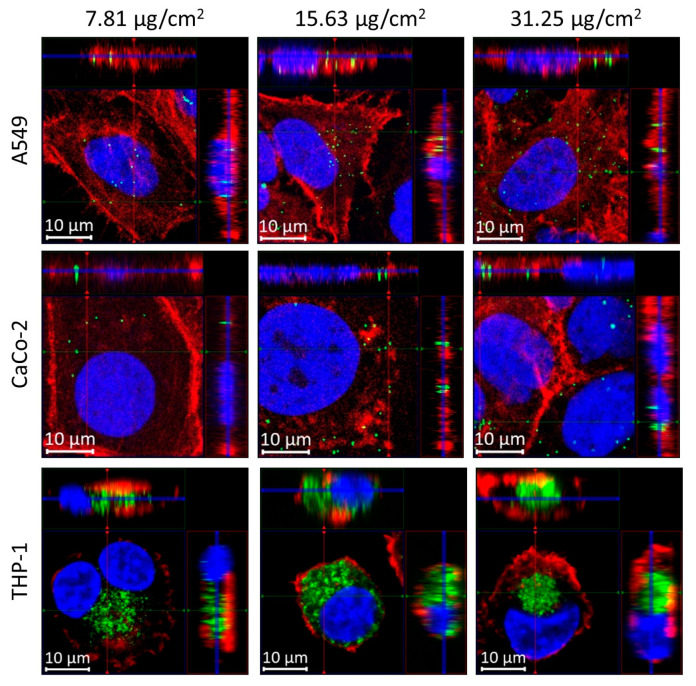
3D-visualization (ortho-projection-view) of polystyrene bead (200 nm) uptake in different cell lines using fluorescence microscopy. For each panel, the images show the xy view in the big bottom-left square at the z position of the blue line shown in the xz (top, projection along the green line in xy) and yz (right, projection along the red line in xy) view. Cells were incubated for 24 h with different concentrations (**first column**: 7.81 µg/cm^2^; **second column**: 15.63 µg/cm^2^; and **third column**: 35.25 µg/cm^2^). **Top row:** A549 cells; **middle row**: CaCo-2-cells; **bottom row**: THP-1 cells. Polystyrene beads show intrinsic dragon green fluorescence (green). Cell nuclei are labelled with DAPI (blue) and actin filaments with phalloidin (red).

Cells were incubated with different concentrations of polystyrene beads with a 200 nm diameter and analyzed after 24 h and after 72 h of incubation (Supplementary Figure S2). Figure 1 shows representative fluorescence images of individual cells of the three different cell lines (A459, CaCo-2, and THP-1 cells) after incubation for 24 h at different concentrations. The green fluorescent polystyrene nanoparticles can be detected in all images inside the cells, as is clearly visible from the 3D ortho-projections at the selected xz- and yz-planes (Figure 1). Larger fields of view (FOV) of detailed images of the different cells with incorporated nanoparticles are presented in Supplementary Figure S3.

**Figure 2:**
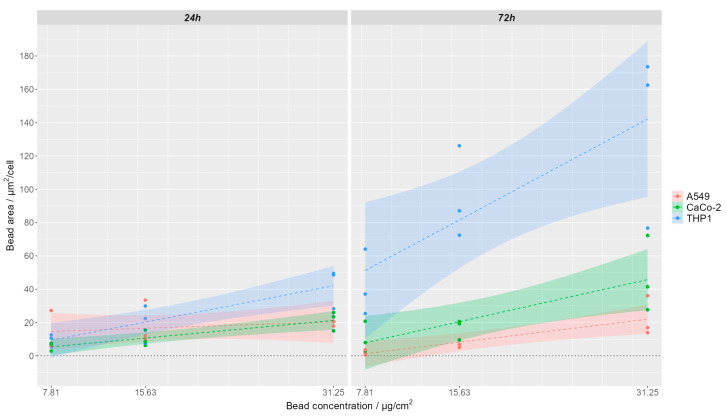
Concentration-dependent uptake of polystyrene (PS) nanoparticles after 24 h (**left**) and 72 h (**right**) incubation time for A549 cells (red), CaCo-2 cells (green), and THP-1 cells (blue)**.** Lines represent linear regression with shaded 95% confidence intervals.

Polystyrene nanoparticle uptake was semi-quantitatively analyzed by determining the area of polystyrene bead-associated fluorescence and normalizing it to the number of cells found in the respective field of view. Example images of the analyzed FOV are shown for one representative replication in Supplementary Figure S4 for 24 h of incubation with nanoparticles and in Supplementary Figure S5 for 72 h of incubation with nanoparticles. The segmented cell area is indicated by yellow lines. Within each of the three replicates, the area of the analyzed field of view was 722,121 µm^2^ (THP-1) or 499,514 µm^2^ (A549, CaCo-2). The respective mean values for the different applied nanoparticle concentrations (7.81, 15.63 and 31.25 µg/cm^2^ culture area) for the different incubation times are depicted per cell line in Supplementary Figure S6, and a linear regression of the concentration-dependent increase of ingested bead area per cell is visualized in Figure 2.

For both incubation time points (24 h and 72 h), higher nanoparticle concentrations in the medium also resulted in a higher uptake of the nanoparticles as indicated by a higher area of polystyrene beads per cell (Supplementary Figure S6). The significance was tested by a two-sided unpaired *t*-test and is marked in Supplementary Figure S6 (* *p* ≤ 0.05). For all tested cell lines, the area of detected beads ingested per cell increased with increasing concentration of PS beads in the cell culture medium (Figure 2, Supplementary Figure S6). This increase of ingested particles in response to the medium concentration was highest for THP-1 cells (Figure 2), probably as a result of the active phagocytotic activity of these cells. Comparing the uptake of nanoparticles in different cell lines, for the two lowest observed concentrations (7.81 µg PS/cm^2^ and 15.62 µg PS/cm^2^), no differences can be seen between the three cell types when looking at 24 h incubation time (Figure 2, Supplementary Figure S6). However, when looking at 31.25 µg PS/cm^2^ for 24 h and for all nanoparticle concentrations at the 72 h incubation time, a higher number of nanoparticles is taken up by the THP-1 cells compared to the A549 and CaCo-2 cells (Figure 2, Supplementary Figure S6). When comparing the nanoparticle uptake within the same cell line for the different incubation time points, we noticed that, for THP-1 cells, there is a significantly higher nanoparticle uptake observed at longer incubation times. For the two epithelial cell lines, A549 and CaCo-2, this trend is not observed.

THP-1cells stimulated over 72 h approximately doubled-to-quadrupled the mean uptake of PS beads per cell compared to a exposition time of 24 h (Supplementary Figure S6c) within one concentration group.

### 3.2. Label-Free 3D Visualization of Intracellular Nanoparticles Using Raman Imaging

**Figure 3:**
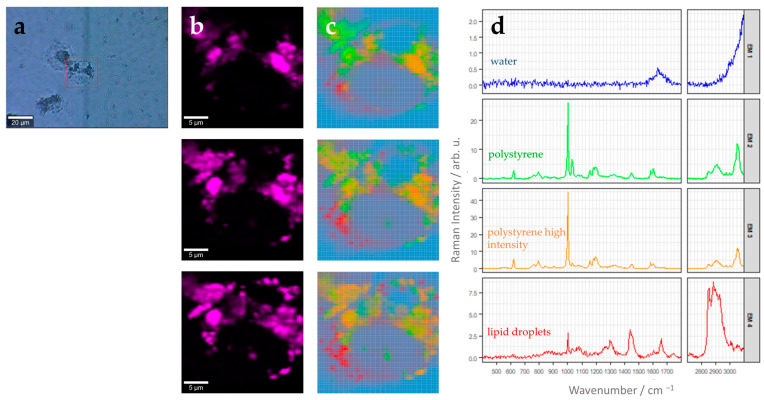
Label-free characterization of polystyrene uptake by THP-1 macrophages using Raman spectroscopic imaging. (**a**) Bright-field image after stimulation for 24 h with 5.6 µg of 220 nm polystyrene beads per cm^2^. The red square indicates the region of interest (ROI) used for Raman image analysis. (**b**) False color Raman images of the ROI in three different z-planes, each 1.5 µm apart. Color codes for relative Raman intensity of the polystyrene aromatic ring breathing mode around 1000 cm^−1^ (width: 25 cm^−1^) (bright pink: high intensities, black: low intensities). Image size: 25 µm × 25 µm with pixel size 0.5 µm × 0.5 µm. (**c**) False color Raman images of the ROIs shown in panel (**b**) after N-FINDR analysis. Respective endmember spectra and color-code assignment is provided in panel (**d**). (**d**) Endmember (EM) spectra corresponding to the image in (**c**). EM1, blue: water/background, EM2, green: polystyrene beads, EM3, orange: polystyrene beads (higher intensity than green polystyrene spectra) EM4, red: lipid droplets. Please note different scaling of the Raman intensity axis, which was chosen to optimally reveal spectral features of each endmember.

The nanoparticle uptake was also visualized inside intact THP-1 cells using label-free Raman spectroscopic imaging. Here, it was not necessary to label the nanoparticles with a fluorophore, and plain polystyrene beads were used to perform uptake experiments. An exemplarily bright field image of THP-1 cells after nanoparticle uptake is shown in Figure 3a. Directly around the cells, a clear region is visible, while further away from the cells, several clusters of nanoparticles can still be detected, indicating that THP-1 cells actively take up particles in their surroundings.

A Raman image scan was recorded in three different z layers from a selected cell. The layers are 1.5 µm apart from each other, spanning a distance of 3 µm. There are different ways to generate Raman false-color images using the multidimensional spectral data available in each pixel of the image. As we are interested in the abundance of the nanoparticles in the cell, we can use characteristic Raman spectral features of polystyrene. Polystyrene has an aromatic ring in the side chain of the polymer and, thus, shows a prominent Raman band at 1000 cm^−1^ originating from the aromatic ring breathing mode (Supplementary Figure S7, Table 1). False-color Raman images after univariate analysis, where the intensity distribution of the Raman band around this wavenumber (1000 cm^−1^) is depicted using an intensity color scale, are shown in Figure 3b. Those images displaying the intensity distribution of the polystyrene Raman mode can provide a first indication of the intracellular distribution of the nanoparticles.

More advanced data-analysis algorithms aim to spectrally unmix the different chemical components present in the hyperspectral image. One popular algorithm to do this is N-FINDR analysis [27]. Figure 3c represents abundance maps of four endmembers in the different z planes of the same cell depicted in Figure 3b. The endmembers were extracted using the N-FINDR algorithm, and the abundances were calculated using NNLS. The corresponding endmember spectra are depicted in Figure 3d. Each endmember spectrum corresponds to a pixel on the hyperspectral Raman image. According to their characteristic Raman bands, the four endmembers can be assigned to water (EM1, blue), polystyrene (EM2, green), polystyrene with a higher intensity than EM2 (EM3, orange), and lipids (EM4, red).

A detailed Raman band assignment is provided in Table 1. The water spectrum shows very little spectral features and a weak broad hump around 1644 cm^−1^, which can be assigned to the H-O-H bending vibration of the water molecules. This endmember is found mainly around the cells. Endmember 4 depicts characteristic vibrational bands found in lipids. In particular, the sharp Raman band around 1656 cm^−1^ originating from C=C stretching vibration, the band around 1442 cm^−1^ from scissoring vibrations of CH_2_ or CH_3_ groups, and the intense Raman band around 2882 cm^−1^ for the antisymmetric =C-H(2) stretching vibration (Figure 3d, Table 1). The endmember spectra characterizing polystyrene (EM2 and EM3, Figure 3d) show many sharp and prominent Raman bands, which can be assigned to vibrations of the aromatic ring (e.g., Raman bands at 621 cm^−1^, 1001 cm^−1^, 1604 cm^−1^, 3057 cm^−1^) and the polymer backbone chain (e.g., 1031 cm^−1^, 1450 cm^−1^, 2913 cm^−1^). It has to be noted that EM2 shows excellent agreement with Raman spectra recorded from pure nanoparticles and with polystyrene reference spectra published earlier (see Supplementary Figure S7). Polystyrene particles are distributed in all z-planes around the cell nucleus.

**Figure 4:**
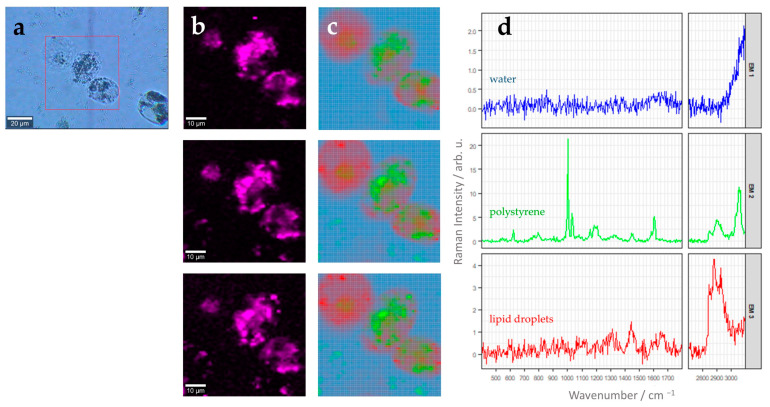
(**a**) Brightfield image of THP-1 macrophages after stimulation for 24 h with 5.6 µg 220 nm polystyrene beads per cm^2^. The red square marks an area of 60 µm × 60 µm, which was characterized by Raman imaging with step size of 1 µm × 1 µm. (**b**) False-color Raman images of the cells representing relative Raman intensity of the aromatic ring breathing band around 1000 cm^−1^ (width: 25 cm^−1^) as color scale (bright pink: high intensities, black: low intensities). Three different z-planes are shown with a spacing of 1 µm. (**c**) False-color Raman images after N-FINDR analysis. Same z-planes as in panel (**b**) are shown. Color code corresponds to the respective endmembers shown in panel (**d**). (**d**) Endmember (EM) spectra corresponding to the image in (**c**). EM1, blue: water/background; EM2, green: polystyrene beads, EM3, red: lipid droplets. Please note different scaling of the Raman intensity axis, which was chosen to optimally reveal spectral features of each endmember.

Similar analysis can also be performed with larger scan areas where more than one cell is present within the field of view. Figure 4 shows a three-dimensional image scan comprising three different THP-1 cells stimulated with the 220 nm polystyrene beads for 24 h. As already discussed above (Figure 3), N-FINDR analysis clearly revealed the presence of polystyrene particles with a distinct endmember (EM1, Figure 4c,d) inside the THP-1 cells. Again, the characteristic Raman spectral fingerprint of polystyrene discussed earlier (Supplemental Figure S7) is clearly revealed in the endmember spectrum (Figure 4d). Polystyrene microparticles are distributed in all z planes around the cell nucleus. However, the uptake slightly varies between different individual cells. In addition, lipid droplets are found throughout the THP-1 cell (EM3, Figure 4c,d), as was also seen in Figure 3. By contrast, THP-1derived macrophages not stimulated with PS nanoparticles showed only endmember spectra of water, lipid droplets, and protein-rich material (see Supplementary Figure S8).

## 4. Discussion

Increasing amounts of microplastics can be found everywhere in the environment, in water, soil and air, also exposing humans and animals to these presumably harmful substances and increasing the potential of microplastic uptake through inhalation or ingestion. Thus, the detection and quantification of microplastics is of the highest interest. In this contribution, we studied the uptake of polystyrene particles by potential barrier cells (A549 lung cells, CaCo-2 gut cells, and THP-1 monocytes/macrophages). Fluorescence microscopy and Raman spectroscopy proved to be suitable methods for detecting the intracellular uptake of polystyrene particles.

Fluorescence imaging requires the use of particles, which carries a fluorescent label. Here, we used Dragon Green dye, which showed an emission maximum in the green (520 nm). In vitro uptake experiments proved that microplastic particles, in our case, polystyrene, are taken up by all used cell lines in all used concentrations (ranging from 7.81 µg/cm^2^ to 31.25 µg/cm^2^ polystyrene beads per culture area). Please note that single fluorescence beads are below the resolution limit and cannot be detected individually. To compare the number of endocytosed particles in different cell lines and at different exposure rates, we chose a semi-quantitative approach by first performing a z-projection and then determining the area showing nanoparticle fluorescence. We are aware that the density of nanoparticles in this area might still be slightly different in different settings. However we are confident that these variations do not contradict the overall conclusions drawn from that approach. Since the highest density of nanoparticles (as judged by the overall fluorescence intensity) seems to exist in THP1 cells, which also show the highest overall uptake, we might probably have underestimated the amount of uptake in these cells as compared to the other cell types.

Here, we show that higher exposure concentrations also resulted in higher polystyrene concentrations inside all cell types for both incubation times (24 h and 72 h). Extended exposure, in our case, 72 h compared to 24 h, resulted in higher intracellular concentrations of particles found in the professional phagocytes, the immune cell line THP-1, but not in the alveolar or intestinal epithelial cell lines, A549 and CaCo-2, respectively. The reason for this observation might be the active search for and uptake of foreign particles by migrating immune cells by phagocytosis. In contrast, the rather immobile epithelial cells might take up any particle that hits them during exposure by pincytosis. Since the cell culture medium was not shaken or removed during the incubation period, no extended further uptake is expected after the initial exposure. Since the epithelial cells also actively divide, the number of particles per cell might even reduce over time since they might be split into the daughter cells. According to the provider (https://www.atcc.org, (accessed on 21 December 2023)), A549 cells have the shortest doubling time of approximately 1 day, while Caco-2 cells would double within 3–4 days. This is in agreement with our observation of a sometimes-slight decrease in accumulated nanoparticles per cell after 72 h of incubation compared to 24 h of incubation. By contrast, THP-1 cells, which were differentiated to macrophage using PMA, are reported to no longer divide [33]. Compared to tissue-resident cells, like the A549 lung cells or CaCo-2 gut cells, uptake by the immune cells as professional phagocytes was higher. This is also expected from their specific function in the body to remove foreign material, such as bacteria or viruses, by phagocytosis, while epithelial cells are expected to engulf material only by pinocytosis [8]. The typical size of bacteria and viruses is on the same order of magnitude as microplastics.

Our findings of the uptake of polystyrene microplastics in the three different cell lines as representatives of three different barriers in the body, i.e., lung, gut, and immune system, align with reports from contamination studies in humans. Here, microplastics of different types can be found in various human tissues, including blood, liver, lung, placenta, kidney, spleen, sputum, and feces [34].

While fluorescence microscopy allows for relatively easy detection, localization, and quantification of microplastics inside the eukaryotic cells, it requires the microplastic particles to be fluorescent. For defined in vitro studies, this poses no difficulty as fluorescently labeled microplastics are available. However, if the uptake of environmental samples is to be considered, a method that can detect intracellular microplastic in a label-free manner is needed. Here, vibrational spectroscopy holds high potential. If sub-cellular spatial resolution is also of need, Raman micro-spectroscopy is a powerful method and was applied in this study. In agreement with the results of the fluorescently labeled polystyrene microplastics, it could be confirmed that THP-1 cells take up significant amounts of (also non-labeled) polystyrene particles. The particles are found in the cytoplasm, where they accumulate. Z-stack images allowed for the visualization of the microplastic particles in three dimensions within the eukaryotic cell in organelles around the cellular nucleus. This agrees with earlier uptake studies with polystyrene particles in A549 lung cells [35,36]. Raman-based analysis also holds the potential to follow the intracellular particle trafficking [37]. The drawbacks of Raman spectroscopic imaging are longer measurement times, making quantitative analysis more time consuming. Therefore, in the current contribution, only qualitative evidence for the nanoparticle uptake is provided. On the other hand, this method offers particular advantages for the analysis of unknown microplastics, as measured spectra can be compared with already known reference microplastic Raman spectra to determine the type of plastic and to be able to prove uptake by cells [38].

## 5. Summary and Conclusions

In this contribution, we provided proof for intracellular accumulation of polystyrene microplastic particles both in professional phagocytic cells, as well as by lung and gut epithelial cells in a concentration-dependent manner. The number of particles found in professional phagocytic cells was higher than in epithelial cells and increased over time, indicating a different uptake behavior in these cell types. Raman micro-spectroscopy proved to be a powerful method for label-free detection of microplastics accumulation, which might be used to detect environmental samples in the future.

## Figures and Tables

**Table 1:** Raman band-assignment endmember spectra found in a THP-1 cell stimulated with polystyrene beads. Spectra are displayed in Figure 3d.

Endmember Spectrum (Color: Assignment)	Raman Band in cm^−1^	Assignment (Based on Literature)
Blue: Water	1644	H-O-H bending vibration [28]
Green/Orange: PS Bead	621	Aromatic ring deformation [29,30,31]
	1001	Aromatic ring breathing [29,30,31]
	1031	C-H deformation vibration [29,30,31]
	1201	C6H5-C vibration [29,30,31]
	1450	CH2 scissoring [29,30,31]
	1604	Ring skeletal stretch [29,30,31]
	2913	Anti-symmetric CH2 stretching [29,30,31]
	3057	C-H stretch of aromatic ring [29,30,31]
Red: Lipid droplets	1302	Twisting vibration of CH2 group [32]
	1442	Scissoring vibration of CH2/CH3 group [32]
	1656	C=C stretching vibration [32]
	2882	Antisymmetric =CH2 stretching [32]
	2929	=CH3 symmetric stretching [32]

## Data Availability

Data are available upon request.

## Supplementary Materials

The following supporting information can be downloaded at: https://www.mdpi.com/article/10.3390/cells13050454/s1. Figure S1: Stepwise procedure of nucleus and polystyrene beads counting in Image J using an example of stimulated THP-1 cells with 25 microgram dragon green fluorescent PS beads over a period of 24 h. Cell nuclei were stained with DAPI. Images were taken with the 20×/NA 0.8 objective of the LSM instrument. Scanned area: 722,120.921 µm^2^; Figure S2: Fluorescence images 24 h and 72 h stimulation time. Polystyrene beads show intrinsic dragon green fluorescence (green). Cell nuclei are labelled with DAPI (blue) and actin filaments with phalloidin (red); Figure S3: Detailed images of A549, CaCo-2 and THP-1 cells stimulated with 200 nm polystyrene beads. Cells were incubated for 24 h with different concentrations (First column: 7.81 µg/cm^2^; second column: 15.63 µg/cm^2^, and third column: 35.25 µg/cm^2^); Figure S4: Fluorescence overview images of A549, CaCo-2 and THP-1 cells stimulated with 200 nm polystyrene beads. Cells were incubated for 24 h with different concentrations (First column: 7.81 µg/cm^2^; second column: 15.63 µg/cm^2^, and third column: 35.25 µg/cm^2^); Figure S5: Fluorescence overview images of A549, CaCo-2 and THP-1 cells stimulated with 200 nm polystyrene beads. Cells were incubated for 72 h with different concentrations (First column: 7.81 µg/cm^2^; second column: 15.63 µg/cm^2^, and third column: 35.25 µg/cm^2^); Figure S6: Graphical representation of polystyrene (PS) nanoparticle uptake after different incubation times for A549 cells (a) CaCo-2 cells (b) and THP-1 cells (c). Data represent mean ± standard deviation of three technical replications. Color codes different polystyrene bead concentration in the well: blue 7.81 µg/cm^2^, green 15.63 µg/cm^2^, orange 31.25 µg/cm^2^. Please note the different scaling of the *y*-axis. Statistical significance (tested by two-sided unpaired *t*-test) is marked as follows: * *p* ≤ 0.05; Figure S7: Raman spectra of polystyrene. Black: Raman spectra of pure polystyrene beads (700 nm). Raman spectra were excited using 532 nm laser wavelengths which was focused onto the sample using a 100× objective (NA 0.75, Zeiss), 37 mW laser power, 100 µm-diameter fiber, 600 grooves/mm; Figure S8: Raman analysis of THP-1 macrophage without nanoparticle treatment; Table S1: Average counted cell number per one mm^2^ for the different cell lines and time points.
