# Supplementary material for: Quantification of Polystyrene Uptake by Different Cell Lines Using Fluorescence Microscopy and Label-Free Visualization of Intracellular Polystyrene Particles by Raman Microspectroscopic Imaging
Source: Cells. 2024 Mar 5;13(5):454. doi: 10.3390/cells13050454 (PMC10931371; doi:10.3390/cells13050454)
Supplement: Supplementary file 1 [file cells-13-00454-s001.zip › cells-2874861-supplementary.pdf]

Supplementary information

Quantification of Polystyrene Uptake by Different Cell Lines Using Fluorescence Microscopy and Label-Free Visualization of Intracellular Polystyrene Particles by Raman Microspectroscopic Imaging

Amelie Roth [1,2], Astrid Tannert [1,3], Nadja Ziller [2], Simone Eiserloh [1,3], Bianca Göhrig [2], Rustam R. Guliev [1], María José Gonzalez Vazquez [1,3], Max Naumann [1], Alexander S. Mosig [4], Sven Stengel [5], Astrid R. R. Heutelbeck [2] and Ute Neugebauer [1,3,6,*]

[1] Leibniz Institute of Photonic Technology, 07745 Jena, Germany
[2] Occupational, Social and Environmental Medicine, Jena University Hospital, 07747 Jena, Germany
[3] Center for Sepsis Control and Care and Department of Anaesthesiology and Intensive Care Medicine, Jena University Hospital, 07747 Jena, Germany
[4] Institute of Biochemistry II, Jena University Hospital, 07747 Jena, Germany;
[5] Department of Neuropediatrics, Jena University Hospital, 07747 Jena, Germany
[6] Institute of Physical Chemistry and Abbe Center of Photonics, Friedrich Schiller University, 07743 Jena, Germany
* Correspondence: ute.neugebauer@med.uni-jena.de

Content

Supplementary Figure S1: Stepwise procedure of nucleus and polystyrene beads counting in ImageJ 2

Supplementary Figure S2: Fluorescence images 24 h and 72 h stimulation time. Polystyrene beads show intrinsic dragon green fluorescence (green). Cell nuclei are labelled with DAPI (blue) and actin filaments with phalloidin (red). 3

Supplementary Figure S3: Detailed images of A549, CaCo-2 and THP-1 cells stimulated with 200 nm polystyrene beads. 4

Supplementary Figure S4: Fluorescence overview images of A549, CaCo-2 and THP-1 cells stimulated with 200 nm polystyrene beads. 5

Supplementary Figure S5: Fluorescence overview images of A549, CaCo-2 and THP-1 cells stimulated with 200 nm polystyrene beads. 6

Supplementary Figure S6 Graphical representation of polystyrene (PS) nanoparticle uptake after different incubation times 7

Supplementary Figure S7: Raman spectra of polystyrene 8

Supplementary Figure S8: Raman analysis of THP-1 macrophage without nanoparticle treatment . 9

Supplementary Table S1. Average counted cell number per one mm^2 for the different cell lines and time points 10

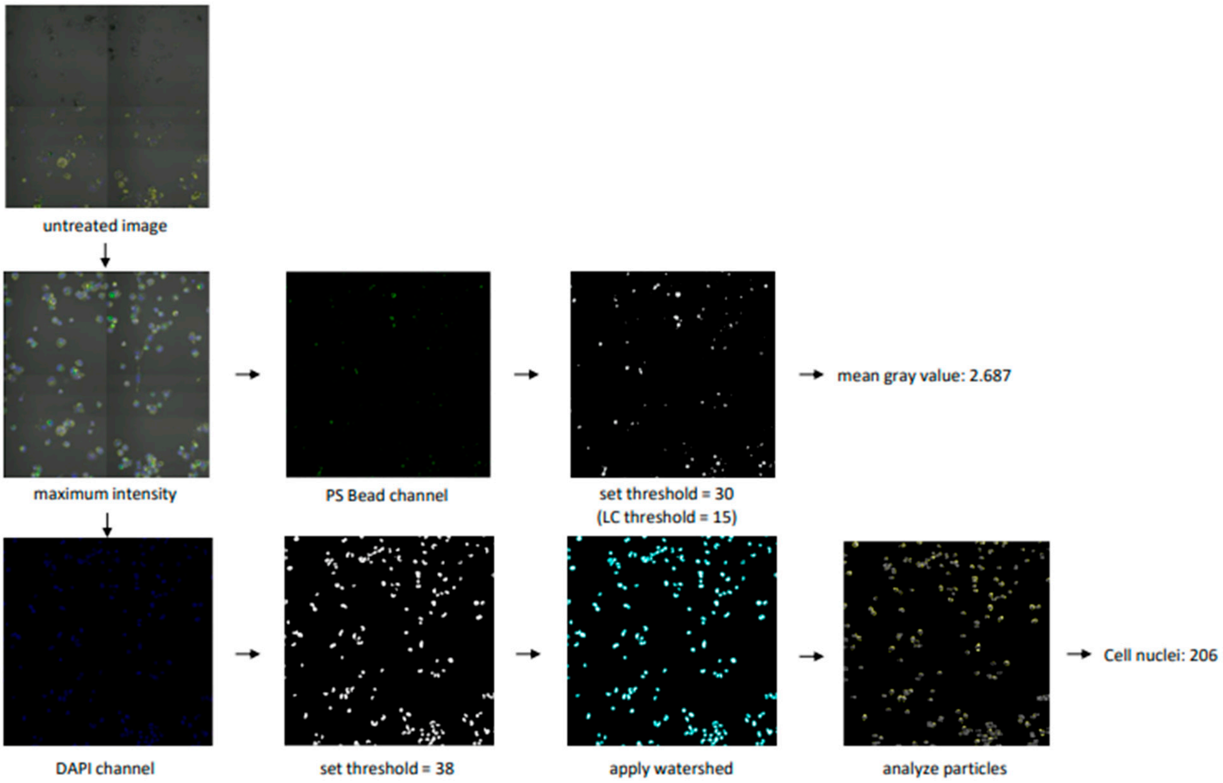

Supplementary Figure S1: Stepwise procedure of nucleus and polystyrene beads counting in ImageJ using an example of stimulated THP-1 cells with 25 microgram dragon green fluorescent PS beads over a period of 24 hours. Cell nuclei were stained with DAPI. Images were taken with the 20x / NA 0.8 objective of the LSM instrument. **Scanned area:** 722120.921 μm^2.

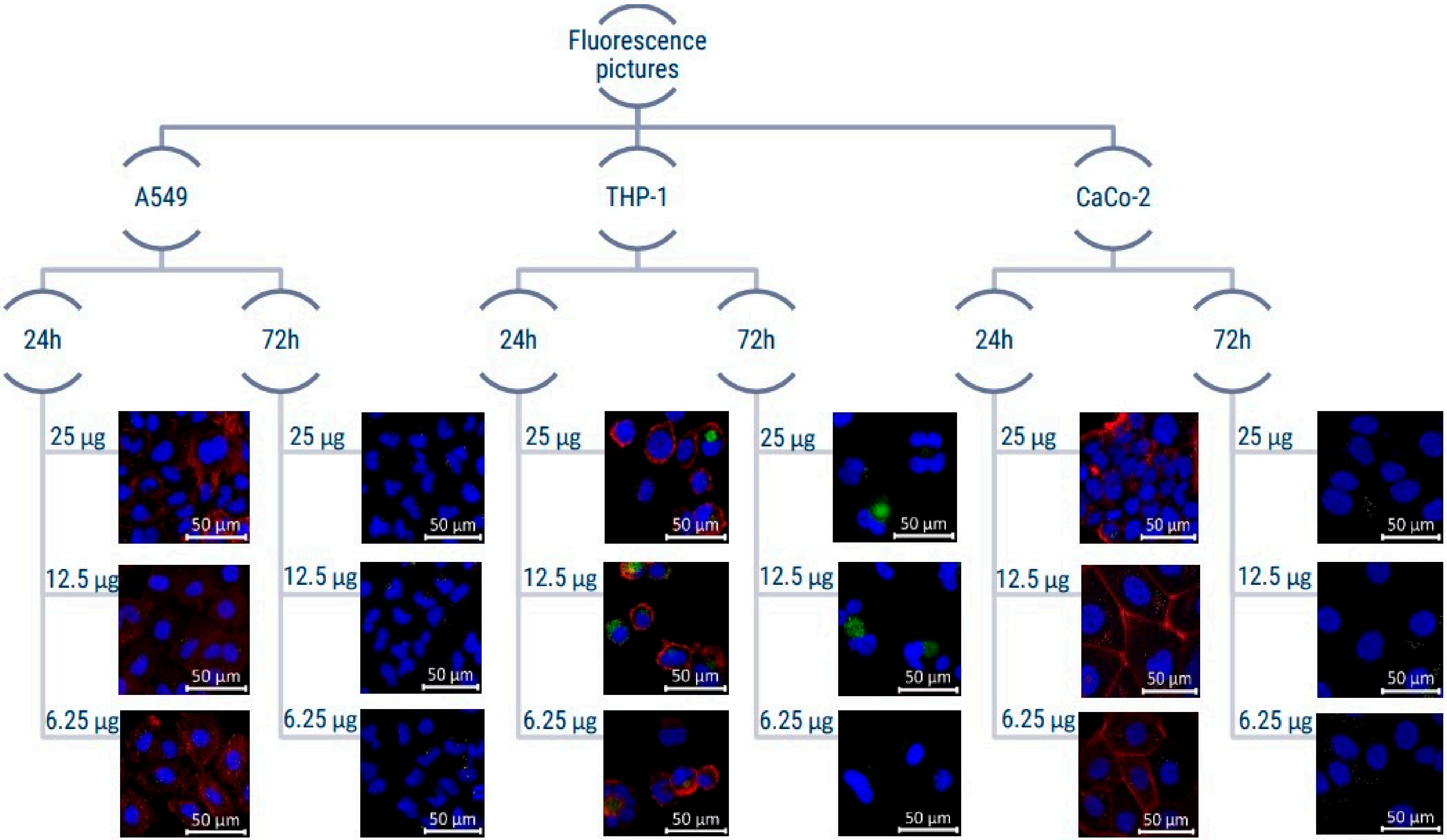

Supplementary Figure S2: Fluorescence images 24 h and 72 h stimulation time. Polystyrene beads show intrinsic dragon green fluorescence (green). Cell nuclei are labelled with DAPI (blue) and actin filaments with phalloidin (red).

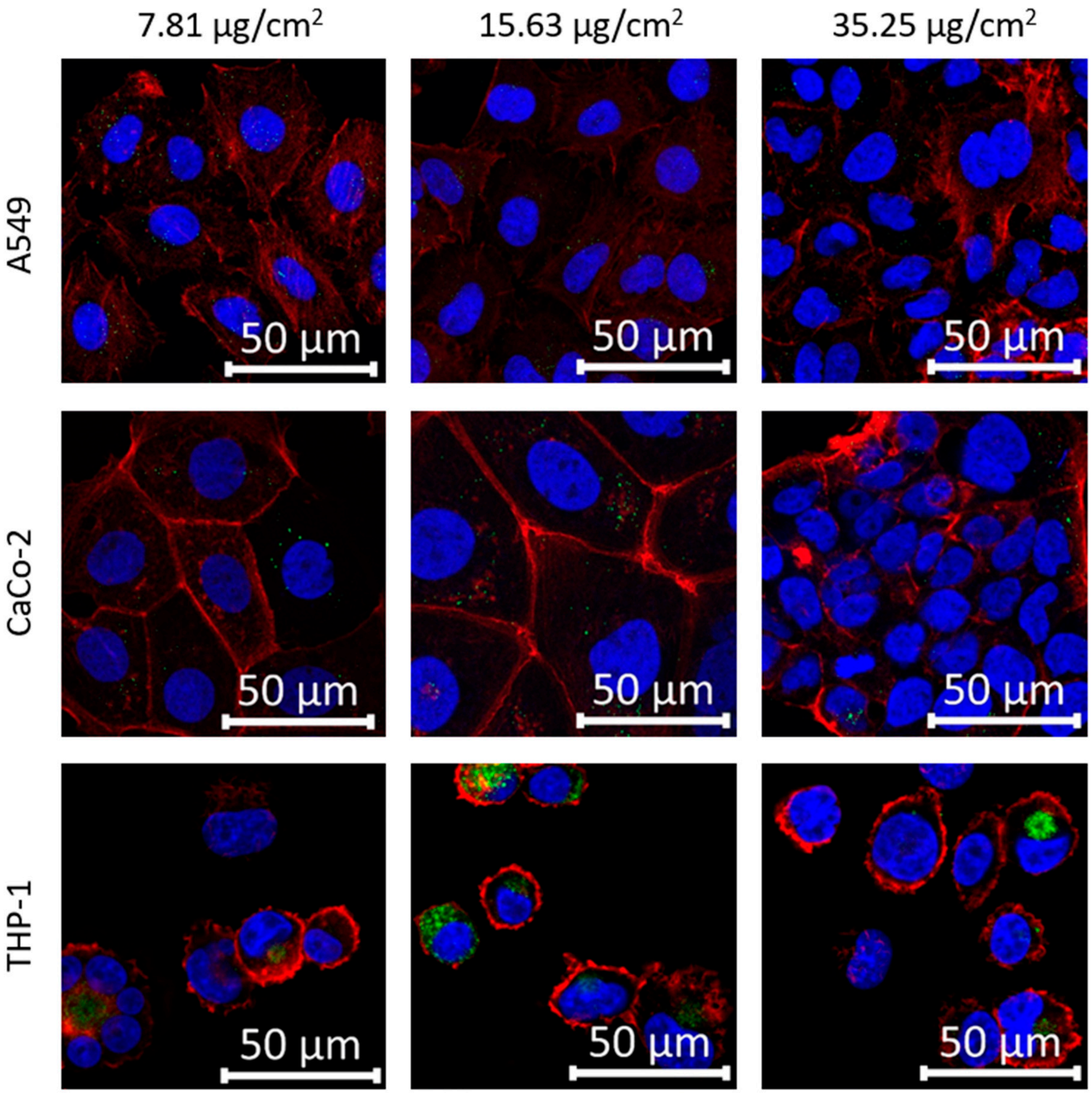

Supplementary Figure S3: Detailed images of A549, CaCo-2 and THP-1 cells stimulated with 200 nm polystyrene beads. Cells were incubated for 24 hours with different concentrations (**First column**: 7.81 µg/cm^2; **second column**: 15.63 µg/cm^2, and **third column**: 35.25 µg/cm^2). **Top row:** A549 cells; **middle row**: CaCo-2-cells; **bottom row**: THP-1 cells and imaged in detail using a 40x objective. Polystyrene beads show intrinsic dragon green fluorescence (green). Cell nuclei are labelled with DAPI (blue) and actin filaments with phalloidin (red).

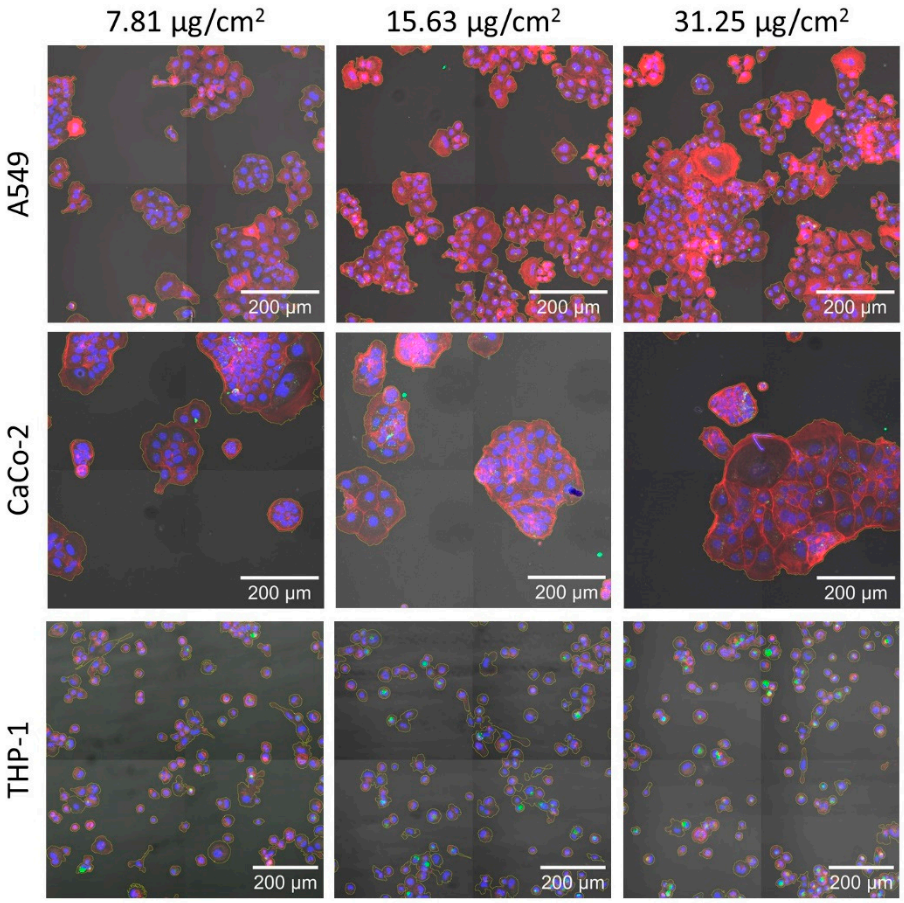

Supplementary Figure S4: Fluorescence overview images of A549, CaCo-2 and THP-1 cells stimulated with 200 nm polystyrene beads. Cells were incubated for 24 hours with different concentrations (**First column**: 7.81 µg/cm^2; **second column**: 15.63 µg/cm^2, and **third column**: 35.25 µg/cm^2). **Top row:** A549 cells; **middle row**: CaCo-2-cells; **bottom row**: THP-1 cells and an area of approximately 0.5 mm^2 was recorded using a 20x objective and 2x2 tile scans. Polystyrene beads show intrinsic dragon green fluorescence (green). Cell nuclei are labelled with DAPI (blue) and actin filaments with phalloidin (red). The segmented cell area is indicated by the yellow lines.

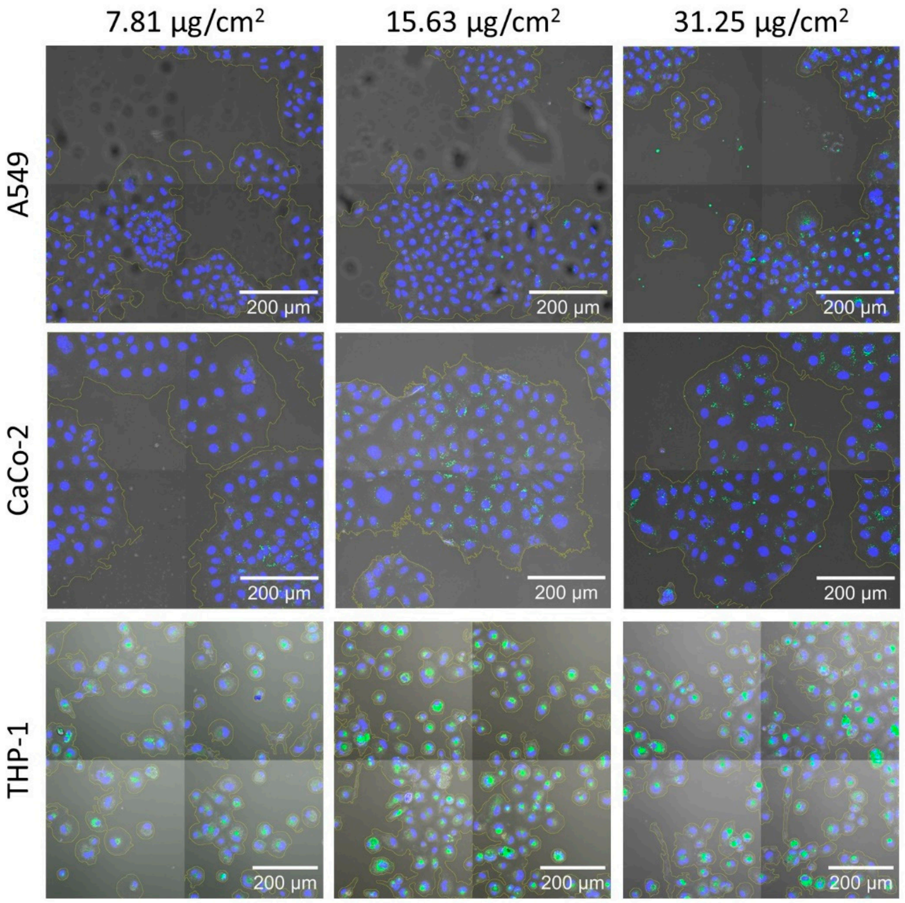

Supplementary Figure S5: Fluorescence overview images of A549, CaCo-2 and THP-1 cells stimulated with 200 nm polystyrene beads. Cells were incubated for 72 hours with different concentrations (**First column**: 7.81 µg/cm^2; **second column**: 15.63 µg/cm^2, and **third column**: 35.25 µg/cm^2). **Top row:** A549 cells; **middle row**: CaCo-2-cells; **bottom row**: THP-1 cells and an area of approximately 0.5 mm^2 was recorded using a 20x objective and 2x2 tile scans. Polystyrene beads show intrinsic dragon green fluorescence (green). Cell nuclei are labelled with DAPI (blue). The segmented cell area is indicated by the yellow lines.

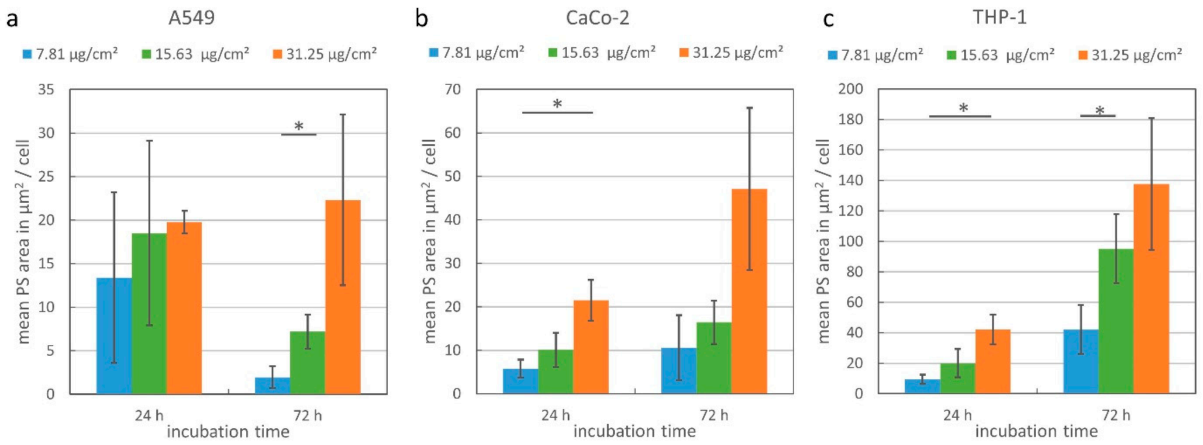

Supplementary Figure S6 Graphical representation of polystyrene (PS) nanoparticle uptake after different incubation times for A549 cells (**a**) CaCo-2 cells (**b**) and THP-1 cells **(c).** Data represent mean ± standard deviation of three technical replications. Color codes different polystyrene bead concentration in the well: blue 7.81 µg/cm², green 15.63 µg/cm², orange 31.25 µg/cm². Please note the different scaling of the y-axis. Statistical significance (tested by two-sided unpaired t-test) is marked as follows: * $p \leq 0.05$.

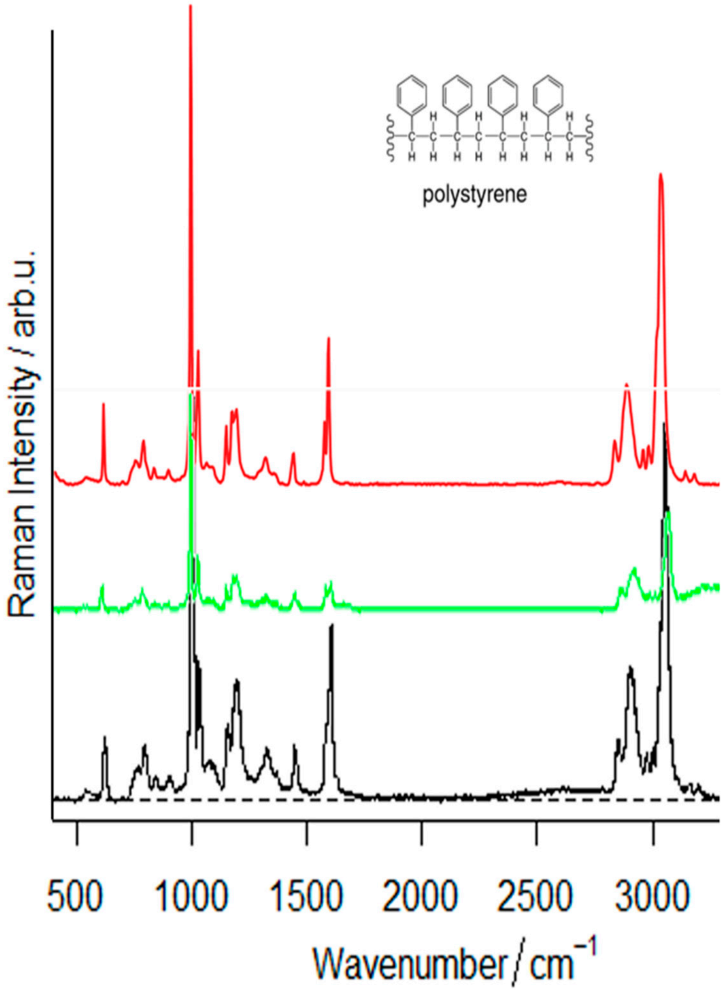

Supplementary Figure S7: Raman spectra of polystyrene. Black: Raman spectra of pure polystyrene beads (700 nm). Raman spectra were excited using 532 nm laser wavelengths which was focused onto the sample using a 100x objective (NA 0.75, Zeiss), 37 mW laser power, 100 µm-diameter fiber, 600 grooves/mm. Green: Endmember spectra of Figure 3 (d) of a THP-1 macrophage after stimulation for 24 hours with 50 µg 220 nm polystyrene beads per well. Red: Spectra visualizes reference literature Raman spectra with the corresponding chemical formula of PS ["Raman Spectrum of Polystyrene (JPG)". Renishaw, https://www.renishaw.com/resourcecentre/en/details/--58670].

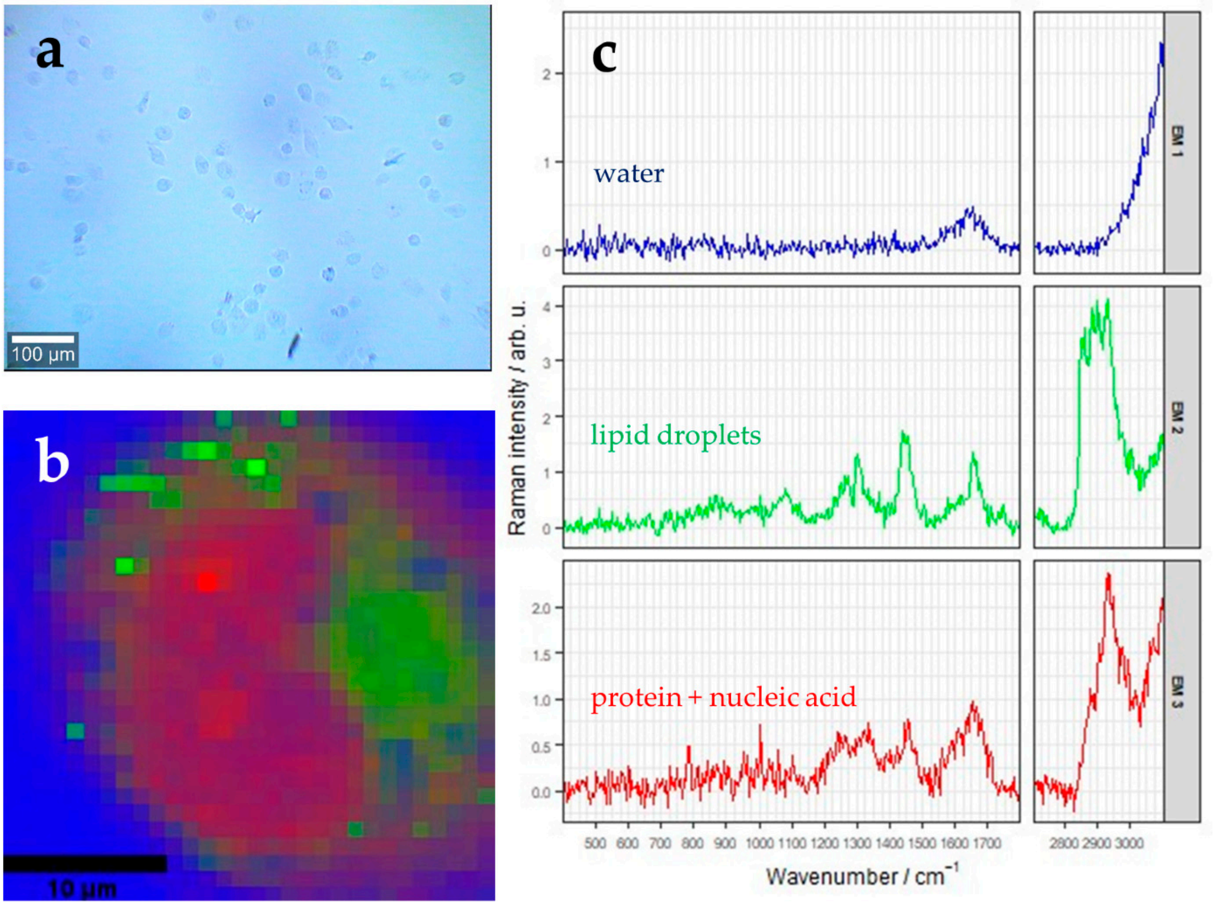

Supplementary Figure S8: Raman analysis of THP-1 macrophage without nanoparticle treatment. (a) Brightfield overview image of non stimulated THP-1 cells (b) False color Raman image of THP-1 macrophages (not treated with PS nanoparticles) after N-FINDR analysis. Image size: 30 µm x 30 µm with pixel size 1 µm x 1 µm. (c) Endmember (EM) spectra corresponding to the image in (b). EM1, blue: water/background, EM2, green: lipid droplets, EM3, red: protein and nucleic acid contributions. Please note different scaling of the Raman intensity axis, which was chosen to optimally reveal spectral features of each endmember.

Supplementary Table S1. Average counted cell number per one mm^2 for the different cell lines and time points. The three different numbers given in each field correspond to the respective average counted cell numbers obtained for the three different replicates. The slash (/) separates the three different replicates.

**Cell line (NP concentration [µg/cm²])**	**24 hours incubation**	**72 hours incubation**
A549 (7.81)	2425 / 386 / 114	5006/ 289 / 484
A549 (15.63)	1650 / 526/ 374	5204 / 242 / 488
A549 (31.25)	1350 / 746 / 378	5224 / 240 / 422
THP-1 (7.81)	166 / 201 / 237	134 / 173 / 565
THP-1 (15.63)	188 / 349 / 266	167 / 301 / 317
THP-1 (31.25)	252 / 548 / 291	159 / 314 / 147
CaCo-2 (7.81)	410 / 146 / 308	982 / 984 / 328
CaCo-2 (15.63)	410 / 214 / 304	482 / 364 / 312
CaCo-2 (31.25)	602 / 232 / 270	1148 / 362 / 244
